# Mesenchymal stromal cells in tumor microenvironment remodeling of BCR-ABL negative myeloproliferative diseases

**DOI:** 10.3389/fonc.2023.1141610

**Published:** 2023-02-23

**Authors:** Enrico La Spina, Sebastiano Giallongo, Cesarina Giallongo, Nunzio Vicario, Andrea Duminuco, Rosalba Parenti, Rosario Giuffrida, Lucia Longhitano, Giovanni Li Volti, Daniela Cambria, Francesco Di Raimondo, Giuseppe Musumeci, Alessandra Romano, Giuseppe Alberto Palumbo, Daniele Tibullo

**Affiliations:** ^1^ Department of Biomedical and Biotechnological Sciences, University of Catania, Catania, Italy; ^2^ Department of Medical-Surgical Science and Advanced Technologies “Ingrassia”, University of Catania, Catania, Italy; ^3^ Department of General Surgery and Medical-Surgical Specialties, A.O.U. “Policlinico-Vittorio Emanuele”, University of Catania, Catania, Italy

**Keywords:** oxidative stress, mesenchymal stromal cells, tumor microenvironment, myeloproliferative cancer, JAK - STAT signaling pathway

## Abstract

Chronic myeloproliferative neoplasms encompass the BCR-ABL1-negative neoplasms polycythemia vera (PV), essential thrombocythemia (ET), and primary myelofibrosis (PMF). These are characterized by calreticulin (CALR), myeloproliferative leukemia virus proto-oncogene (MPL) and the tyrosine kinase Janus kinase 2 (JAK2) mutations, eventually establishing a hyperinflammatory tumor microenvironment (TME). Several reports have come to describe how constitutive activation of JAK-STAT and NFκB signaling pathways lead to uncontrolled myeloproliferation and pro-inflammatory cytokines secretion. In such a highly oxidative TME, the balance between Hematopoietic Stem Cells (HSCs) and Mesenchymal Stromal Cells (MSCs) has a crucial role in MPN development. For this reason, we sought to review the current literature concerning the interplay between HSCs and MSCs. The latter have been reported to play an outstanding role in establishing of the typical bone marrow (BM) fibrotic TME as a consequence of the upregulation of different fibrosis-associated genes including PDGF- β upon their exposure to the hyperoxidative TME characterizing MPNs. Therefore, MSCs might turn to be valuable candidates for niche-targeted targeting the synthesis of cytokines and oxidative stress in association with drugs eradicating the hematopoietic clone.

## 1 Introduction

Chronic myeloproliferative neoplasms (MPNs) might be defined as clonal hematopoietic stem cell (HSC) disorders characterized by an aberrant proliferation of myeloid lineages (1). Furthermore, according to the 2008 World Health Organization Classification Scheme, MPNs are classified by as BCR-ABL1-negative neoplasms (2–5). MPNs encompass a spectrum of clonal hematological disorders, including three main clinical entities: polycythemia vera (PV), essential thrombocythemia (ET), and primary myelofibrosis (PMF) (6). These myeloid malignancies arise due to acquired somatic stem cell lesions affecting calreticulin (CALR), myeloproliferative leukemia virus proto-oncogene (MPL), and the tyrosine kinase Janus kinase 2 (JAK2), usually displaying a valine-to-phenylalanine mutation at 617 (JAK2V617F) (7). This turns to be the most prevalent mutation associated with 95% of PV, 60% of ET and PMF, while CALR and MPL mutations are mainly associated with ET and MF (about the 50% of patients) (8–11). Even though the 10% of MPNs patients are triple negative for these mutations, ET and MF patients may harbor a noncanonical mutations in JAK2 or MPL with its subsequent clonal evolution (12). CALR plays an essential role in programmed cell death induced by oxidative stress (13–16). It is an endoplasmic reticulum chaperone acting in the regulation of protein folding and Ca**^2+^** homeostasis (17) by controlling cellular stress responses (18–20). Increased CALR levels enhance cell sensitivity to hydrogen peroxide (H**_2_** O**_2_**)-mediated cytotoxicity (21). Although the mechanism of action triggered by mutated CALR has not been elucidated yet, its role in inflammation has been associated with its oncogenic action, since it works as an autocrine growth factor in synergy with MPL. Corroborating this scenario, several studies showed that CALR mutations drive oncogenic transformation in a MPL-dependent manner, eventually stimulating JAK-STAT signaling (22–25). For these reasons, CALR, MPL and JAK2V617F, despite they are usually mutually exclusive, have been defined as “driving mutations”. “Founding mutation”, on the other hand, are still unknown (26). Several studies reported MPNs propensity to progress through different disease stages starting from PV or ET towards an aggressive secondary myelofibrosis (MF), finally leading to fibrosis, osteosclerosis, and extramedullary hematopoiesis (27–29). Besides any classification, accumulating evidence suggests that MPNs may be considered as a valid “human inflammation model” for cancer development due to JAK-STAT and NFκB hyperactivation, eventually leading to uncontrolled myeloproliferation and pro-inflammatory cytokines secretion. The most abundant pro-inflammatory cytokines involved in the alteration of hematopoietic TME include platelets-derived growth factor (PDGF), interleukin-1 (IL-1), lipocalin and fibrogenic transforming growth factor-beta (TGF-β). The latter plays a multitude of roles in osteosclerosis and myeloproliferation, promoting Bone Marrow (BM) fibrosis, microvessel density and suppressing physiological blood cell development (30–32). The JAK2V617F mutation has been correlated with a prominent redox alteration since the huge amount of reactive oxygen species (ROS) levels, resulting from an unbalanced H_2_O_2_ ratio following misbalanced catalase concentration (33). The genesis of a steady inflammatory stream ultimately induces a chronic oxidative stress state with elevated ROS levels in the BM niche (34). In such highly oxidative environment, cellular and extra-cellular components need to form a continuum to maintain the balance between biological processes involving HSCs and Mesenchymal Stromal Cells (MSCs). While BM-HSCs are mostly kept in a quiescent state, MSCs differentiate into several cell subpopulation of mesodermal origin, such as adipocytes, chondroblasts, and osteoblasts (OBs) (35, 36). Anatomically, MSCs may be found in various structures, including BM, adipose tissue, and umbilical cord. Inside BM-TME, MSCs are mainly involved in intercellular crosstalk and proliferation (37). As precursors of BM stromal cells, they are thought to play a pivotal role in the pathophysiology of hematological diseases including MPNs. Within this pathological condition, a significant difference between patient-derived and donor-derived MSCs has been described (38). The interplay between MSCs and HSCs in conditioning each other also concerns HSCs-MSCs-lineage (39). Thus, during ROS accumulation, the balance between the two populations is impaired, and it eventually sets the stage for HSCs to evolve

perpetuating vicious circle generates ROS, in turn establishing a prooxidative and inflammatory microenvironment. To escape from these non-permissive conditions, the Suppressors of Cytokine Signaling (SOCS) is activated and binds JAKs and ensures an arrest of the whole inflammatory process (40–42). Another intriguing survival pathway in MPNs has been described by Forte and coworkers, suggesting the contribution of Nestin**^+^** BM-MSCs in acute myeloid leukemia (AML) development and chemoresistance *in vivo* (43). In particular, it has been found that GSH-dependent antioxidant pathways hold as key role in the BM-MSCs crosstalk and represents a potential target for adjuvant therapies in MPNs. It has been also found that Nestin+ niches are reduced in humans or mice with MPNs (43). Despite the existence of different pharmacological strategies against MPNs, including interferon-alpha2 hydroxyurea and statins, patients have higher probability to experience autoimmune issues or the risk of Acute Myeloid Leukemia transformation (44). With the introduction of JAK1/2 inhibitors, such as Ruxolitinib, immune system is potently suppressed, constitutional symptoms are decreased together with pro-inflammatory cytokines burden in BM-TME (45). Nowadays, the main issue with JAK-STAT inhibition is related to the poor Ruxolitinib antioxidant capacity (46). Bjørn et al., evaluated the effect of Ruxolitinib in producing superoxide radicals and H_2_O_2_ by HSCs-derived monocytes in blood samples from patients with MF together with DNA damage. The production of superoxide was significantly decreased during treatment, but no influence on the generation of H_2_O_2_ or the global level of oxidatively altered DNA was found. As the pro-inflammatory cytokine TGF-β plays a central role in MF genesis, and the effect of TGF-β on ROS concentrations has been evaluated in recent studies (47, 48). Results demonstrated that TGF-β administration increases expression levels of a specific miRNA, decreasing SOD2 action, and eventually promoting ROS increase. The recent employment of galunisertib, a small molecule antagonist of the TGF-β receptor type 1 (TGF-βR1) with possible antineoplastic action decreased miRNA expression and ROS increase while resulting in re-established SOD2 activity decreasing the altered oxidative stress state (49). Considering the vicious cycle between chronic inflammation and ROS overproduction, we herein will discuss the role of ROS in MPNs pathogenesis, together with the role of JAK2V617F+ neighboring BM healthy MSCs, as tumor own ability to adapt to BM-TME to manage chronic oxidative stress.

## 2 Role of MSCs in fibrosis

**Figure 1:**
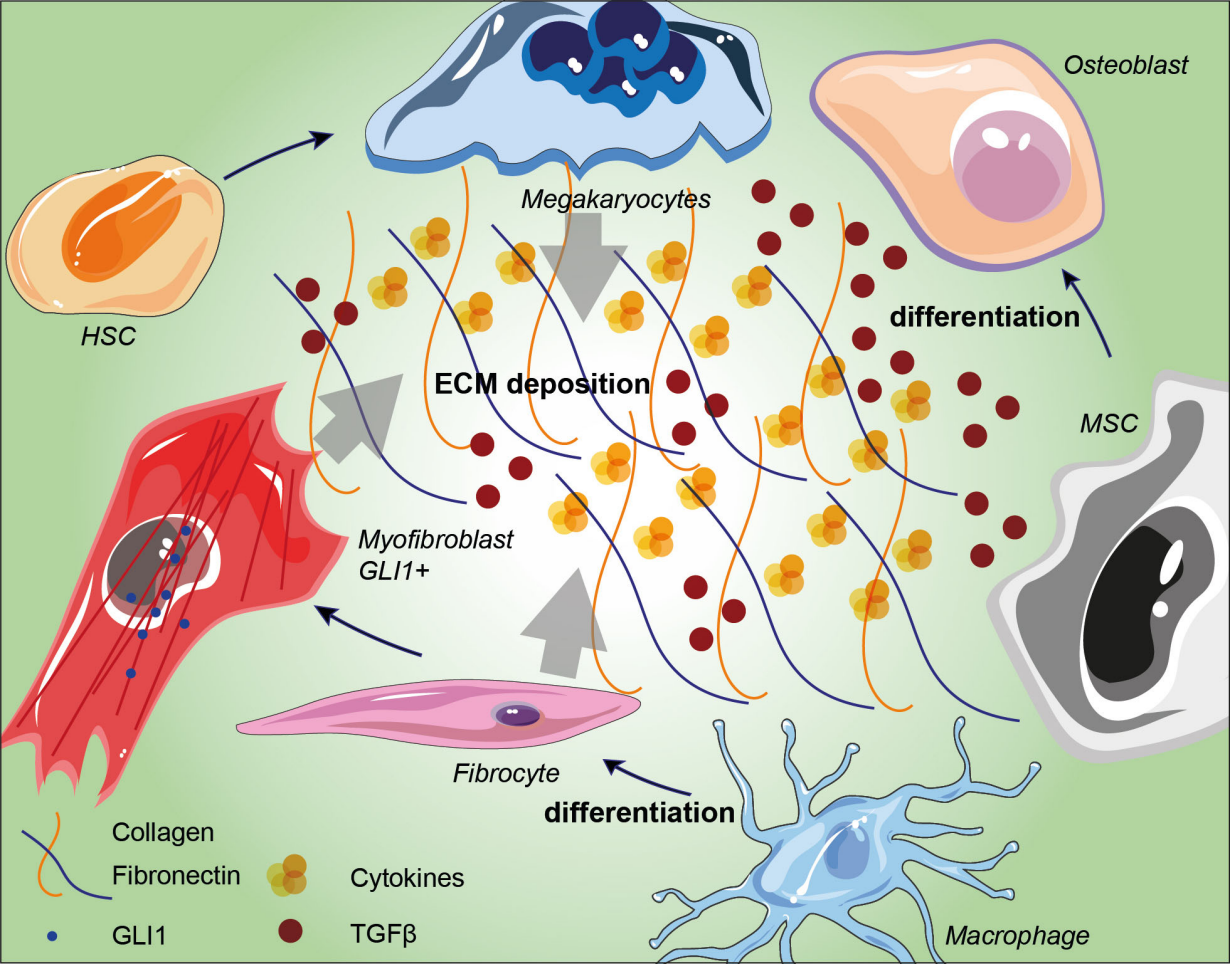
Schematic representation of BM niche fibrosis-driving cell populations and their involvement in extracellular matrix deposition. Arrows indicate the influence of megakaryocytes, fibrocytes and GLI1^+^ myofibroblasts in conditioning ECM in BM niches. TGF-β accumulation induces MSC differentiation into osteoblast and its overall accumulation within the ECM induces ROS production and bone remodeling. HSC: hematopoietic stem cell; MSC: mesenchymal stromal cell; ECM: extracellular matrix; TGF-β: transforming growth factor-beta.

The plethora of stromal cells in a normal BM niche suggests that distinct stromal subtypes have specific roles, not only in normal hematopoiesis but also in fibrosis (50, 51). MF was thought to be a reactive phenomenon caused by the interaction between malignant hematopoiesis and BM-TME mediated by profibrotic cytokines (52). In this context a breakthrough work has been recently published by Schneider’s group. Here, the authors performed a spatial RNA-seq analysis to depict a scenario in which two distinct MSC populations (MSC-1 and MSC-2) are the main drivers of BM fibrosis. The progression towards this phenotype was marked by overexpression of the alarmin complex S100A8/S100A9, which once targeted by a specific inhibitor improved the overall MPN and fibrosis status (53). By the abovementioned genetic fate tracing experiments, together with single-cell RNA-Seq data of the BM niche cell populations, it was possible to identify five major fibrosis-driving MSCs populations: i) GLI1+ myofibroblasts; ii) Leptin receptor (LepR)-; iii) platelet-derived growth factor receptor α (PDGFR-α)-; iv) vascular cellular adhesion molecule 1 (VCAM1)- and v) Nestin-expressing MSCs. Besides their role in fibrosis, these populations also downregulated the expression of key HSC-supporting factors while they upregulated fibrosis-related genes (54). GLI1**^+^** myofibroblasts together with activated cancer-associated fibroblasts (CAFs), play an important role in BM fibrosis genesis (55) and are assumed to be the major sources of new matrix components as well as to reorganize the extracellular matrix (**Figure 1**) (56). This population is characterized by the expression of cell surface antigens typical of the hematopoietic lineage, such as CD45 and CD11b, and also matrix proteins as type I-III collagen and fibronectin. in this context, recent evidence depicted the involvement of IGFBP6/sonic hedgehog (SHH)/Toll-like receptor 4 (TLR4) axis in the TME alterations (57). This cascade has been reported to promote MSCs CAF transition upon IGFBP-6 signaling stimulation (58). Despite high levels of collagen expressed by activated fibrocytes, the extracellular space in their immediate proximity contains few collagen particles and no collagen fibers, thus suggesting that collagen fibers in MF are not polymerized by megakaryocytes’ LOX2 (59). To further investigate possible player within this context, several mice model have been established, which however usually lack in mirroring patients’ condition (32). ASXL mutations, for instance, accelerate BM fibrosis by reprograming the fibrosis-driving potential of hematopoietic cells to fibrocytes, and further confirm that neoplastic fibrocytes are the major contributors to BM fibrosis. Regarding BM fibrosis, GLI1**^+^** myofibroblasts are highly active and contractile cells, characterized by dense rough ER, collagen secretion granules, and α-smooth muscle actin (α-SMA) expression (60). Differential gene expression analysis demonstrated that megakaryocyte-associated genes were significantly up-regulated by these cells. Interestingly, among them CXCL4, cytokine-cytokine receptor interaction pathway, has been depicted as crucial in establishing a fibrotic state (61) and in regulating HSCs (60). Approximatively half of all myofibroblasts derive from the GLI1 migrate into the hematopoietic marrow and differentiate into matrix-producing cells (60). Data indicate that targeting GLI proteins inhibits GLI1+ cell proliferation and myofibroblast differentiation, which results in reduced fibrosis and improved organ function (62). Intriguingly, GLI1+ and LepR+ cells are CD45– non-hematopoietic cells, which indicates that their differentiation into myofibroblasts is a distinct process and is independent of monocyte-derived fibrocytes (55). Arranz et al., showed that BM Nestin+ MSCs, which are innervated by sympathetic nerve fibers, regulate normal HSCs and that abrogation of this regulatory circuit is essential for MPN pathogenesis. It has been demonstrated in MPN and also in other tumors, that MSCs expressing the marker Nestin directly support cell survival and chemoresistance by increasing oxidative phosphorylation (OXPHOS) and simultaneously provide with key antioxidant tools necessary to balance ROS levels during chemotherapy (63–65).

## 3 The role of MSCs in osteosclerosis

**Figure 2:**
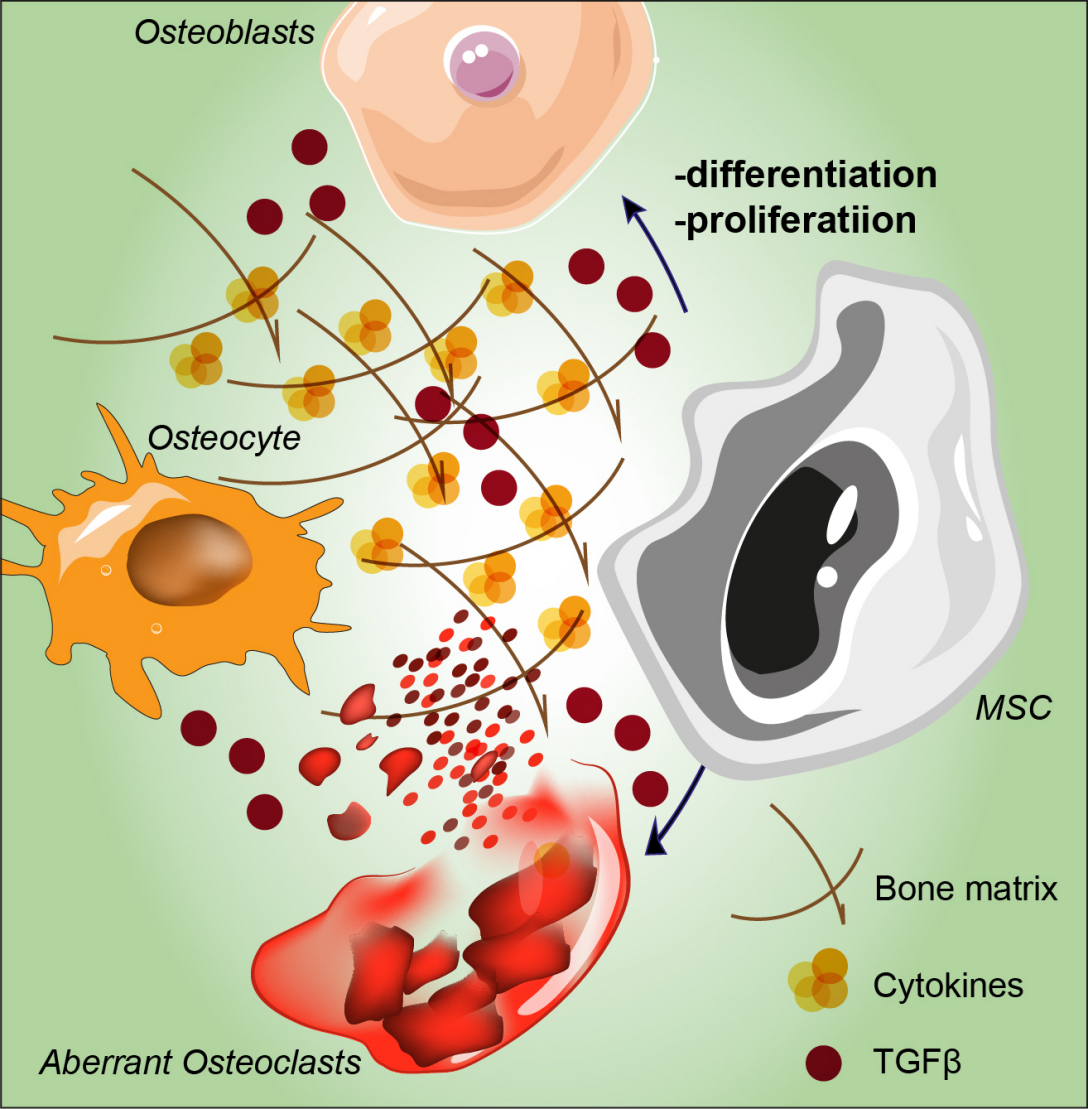
Schematic representation of MSC proliferation and differentiation abnormalities stimulated by tumoral hematopoietic cells. Aberrant osteolytic activity mediated by osteoclasts leads to bone remodeling and to altered osteocytes, overall contributing to the induction of osteosclerosis. MSC: mesenchymal stromal cell; TGF-β: transforming growth factor-beta.

In addition to BM fibrosis, up to 70% of MF patients develop osteosclerosis, a well-established indicator of poor prognosis. Recently, Avanzini and colleagues reported genetic and functional aberrations of BM-MSCs in MPNs and showed that MSCs exhibit decreased proliferative abilities as well as decreased osteogenic capacities also confirmed through *in vitro* ed *in vivo* experiments (32). The direct effect of these alterations is a shift in the balance between osteoblastogenesis and osteoclastogenesis, creating an osteosclerotic state (66). Bone modifications are hallmark of PMF since they represent one of the direct results of BM disruption. Osteosclerosis remains the most common bone change, which represents a pathological event characterized by increased bone density and abnormal hardening (66), and its pathogenesis is still largely unknown. In recent years, an increasing number of studies point towards an important involvement of the MSCs niche in osteosclerosis. Firstly, osteosclerotic regions are produced by the irregular thickening of bone trabeculae, new bone shaping and consequent bone volume growth, secondly, increased BM activity in some regions, such as the vertebral column, pelvis or proximal segments of long bones, remain the most affected by such alterations (67). The physiological bone morphology and functionality are strictly dependent on the accurate setting of the marrow osteoblastic niche as well as the balance between mature bone tissue, endosteum and central BM (68). As part of the BM niche, MSCs support hematopoiesis and restore the differentiated compartment of OBs during tissue growth and turnover (69–71). Tumoral hematopoietic cells stimulate MSCs to proliferate and to adopt an abnormal differentiation program that results in the overproduction of functionally altered OBs lineage cells, which accumulate in the BM cavity as inflammatory myelofibrotic cells (**Figure 2**) (72). Park et al. (73), showed that a subset of Nestin+ MSCs found *in vivo* are able to replace short-lived mature OBs to maintain homeostasis and respond to bone injury. MF osteosclerosis is thought to be induced by overstimulated MSC-derived OBs or impaired bone resorption. While OBs differentiation is also due to TGF-β1 release, in turn inducing the expression of bone morphogenetic protein (BMP), osteoclasts (OCs) are increased and they show an impaired osteolytic activity, eventually distorting bone remodeling and contributing to the induction of osteosclerosis (74). The development of OCs is highly skewed towards proliferation in both primary and secondary MPN-associated MF. Veletic and coworkers demonstrated that neoplastic monocytic progenitors retain their aberrant genetic constitution, even after full differentiation into mature OCs (74). However, fusion of such progenitors seems to be profoundly impaired, and MF OCs are unable to fully acquire the phenotypical features associated with efficient bone resorption, particularly multinucleation and the development of actin-rich structures. At least to a certain extent, this process happens independently of the non-malignant MSCs, although it seems to be impacting the OBs as well. Evidence demonstrates that even though MF OCs are hyperproliferative, their function is intrinsically suppressed due to the inherited neoplastic burden, which in turn contributes to the osteosclerotic dysplasia of the MPN-affected BM (74). Schepers and coworkers showed that OBCs derived from multipotent stromal cells, expand in the presence of malignant hematopoietic cells, which results in matrix production and trabecular thickening (75). Under osteogenic differentiation conditions, MSCs from PMF patients showed an increased capacity to mineralize extracellular bone matrix *in vitro* and to form new bone *in vivo* in immunodeficient mice (32). Furthermore, novel pharmacological approaches are on their way to improve the current strategies in fighting MPNs progression. In this context it has been reported that targeting IL-1β by using a specific antibody might decrease reticulin fibrosis and osteosclerosis in a preclinical JAK2-V617F MPN mouse model. For this reason, a combination therapy with JAK1/2 inhibitor might represent a future direction for MPN therapeutic approach [86].

## 4 The role of oxidative stress in MPN-MSCs

As described above, the perpetual generation of an inflammatory stressing state, leads to the establishment of an optimal environment for mutations accumulation, including MSCs. Allegra et al. reported that by stimulating p38–MAPK, AKT/mTOR and JAK/STAT in progenitors and leukocytes it causes a condition of chronic oxidative stress with increased concentrations of ROS in the BM, eventually triggering germline mutations (40). Such not permissive condition has been correlated with MPN onset. Supporting this data, increased ROS levels produced by TME components the stem cell clone itself constantly produces inflammatory components in the BM. These elements further increase clonal expansion, thus leading to a positive feedback loop (40). In order to characterize the role of altered BM-MSCs during inflammation, Desterke et al. collected information from PubMed and gene databases and crossed these with the gene expression profile of BM-MSCs performed in PMF patients (76). Several altered pathways were identified including oncostatin M and TGF-β signaling triggering misregulated DNA damage, senescence, and autophagy. This has been also confirmed in PV and ET where chronic inflammation in BM-TME induces a hypersensibility of MSCs to inflammatory molecules participating in creating the previous mentioned vicious circle (77). Since TGF-β1 and the subsequent oxidative stress are key regulators for DNA methylation in cancer (78), it has been reported that PDGF-β gene is hypomethylated, thus correlating with a poor prognosis, eventually harboring a significant fibrosis grade (76). Halogenated cytosine residues are indeed one of the most common DNA damage signature mediated by inflammation (79–81). These inflammation damage products have been detected in human leukocytes where the methyl-binding proteins are not able to distinguish methylated and halogenated DNA; thus, DNA methyltransferase (DNMT) 1 could be deceived and they lead to the accumulation of these analogues within the genome (82–86). An initial halogenation, triggered by inflammation, could direct methylation of the complementary DNA strand, eventually resulting in heritable alterations in genome methylation pattern.

## 5 Conclusions and future directions

Herein, we reviewed the regulatory properties of MSCs in immune-mediated or inflammatory conditions, shed light on the fact that MPNs are diseases sensitive to ROS overload together with the central role of the innate immune system in the modulatory effects of MSCs. The pro-inflammatory status induced by oxidative stress increases the risk of malignant transformation. Many aspects concerning the relationship between chronic myeloproliferative diseases, genetic alterations, and inflammation were recently clarified. MPNs are characterized by increased generation of one or more forms of myeloid cells, together with the impairment of neighboring healthy MSCs (40). The excessive ROS accumulation give rise to overstimulated fibrocytes and OCs leading to the onset and progression of disease (87). Inflammatory cytokines are also capable of influencing disease onset as well as its evolution and prognosis. This altered state is also sustained by platelets and megakaryocytes which participate in inflammation and immunity (88). The employment of antioxidants could be advantageous in the treatment of MPNs, since Ruxolitinib has little antioxidative capacity. Furthermore, MPNs patients usually undergoes blood transfusion. The iron overload resulting upon this step might also enhance ROS accumulation (65, 89). For this reason, in the latest research lines has been reported that the usage of N-adenyl-cysteine (NAC) decreases bone marrow fibrosis in patients with MPN and could potentially aid with HSCT engraftment (90). This work might potentially open the path towards the inclusion of antioxidant drugs in the fight against MPN progression. Recognizing the genetic and external elements that participate in the myelofibrotic evolution of MPNs is of crucial importance for early detection as well as to initiate therapies inhibiting or reversing disease development in MPN patients. The fact that genetic ablation of GLI1+ cells abolish BM fibrosis and restores hematopoiesis indicates that GLI1+ MSCs are a promising therapeutic target (53, 60). Considering the cross-influence between the BM extracellular matrix composition and the proliferation/differentiation capability of HSCs, the stimulating issue concerning the impact of stromal cell alterations on hematopoiesis needs to be elucidated. By being “bad stromal cells”, MSCs take entirely part in the “bad seed in bad soil” concept and strengthen the importance of stromal cells in the development of a neoplasia. Therefore, MSCs from patients are good candidates for niche-targeted therapies that, in association with drugs eradicating the hematopoietic clone, would improve patient treatment. In this scenario, novel therapeutic targets are stepping in front. It has been recently reported that MF patients overexpress the cyclin dependent kinase 6 (CDK6) which might turn to be a novel target. Corroborating this idea, the authors show an enhancement of the Ruxolitinib effect in synergy with CDK6 inhibitor Palbociclib, significantly reducing leukocytosis, splenomegaly, and bone marrow fibrosis in Jak2V617F and MPLW515L murine models. However, the improvement of MF-related animal model is nowadays a crucial step to dissect the effect of novel approaches on immune cells and the mesenchymal counterpart in the BM *milieu*. To develop animal models that appropriately address the complex interplay among HSCs, MSCs and immune system in TME where these cells execute their regulatory function, becomes extremely important. In particular, it may be helpful to dissect the molecular pathway involved in the generation of anti-inflammatory cells, *in vitro* and that result may help to design relevant immunocompetent animal models in order to enhance the antioxidant capability of MSCs to positively counterbalance the negative effect of the oxidative damage. From the MSCs point of view it could be possible to manage the mechanisms through which MSCs may promote or suppress tumor progression and the possible tumor-promoting activity of MSCs may be useful in choosing the right mesenchymal population based on the specific cancer type with a successful application in patients. As the main goal to be achieved is to operate on the pathways that control the synthesis of cytokines, oxidative stress and genome instability as well, it may be suggested that acting on the inflammatory state as a therapeutic approach in MPNs could be effective in reducing the possibility of leukemic progression and onset of complications. Nevertheless, this promising property of MSCs, independently on their HSC-supporting capacity and the immunomodulatory effect, warrants extensive and deeper studies.

## Author contributions

Conceptualization, EL, SG, CG, NV, and DT. Writing—original draft preparation, EL, SG, CG, NV, GM, and LL. Writing—review and editing, CG, NV, RP, RG, DC, GL, FD, AR, GM, AD, GP, LL, and DT. Supervision, CG, NV, RP, RG, GM, GL, FD, AR, GP, and DT. Project administration, EL, DT, and NV. All authors contributed to the article and approved the submitted version.

## References

[1] Paule R Ponsoye M Gueutin V Deray G Izzedine H. Myeloproliferative neoplasms related glomerulopathy. Rev Med Interne (2013) 34(6):369–72. doi: 10.1016/j.revmed.2012.12.013 23357690

[2] Tefferi A Thiele J Vardiman JW. The 2008 world health organization classification system for myeloproliferative neoplasms: order out of chaos. Cancer (2009) 115(17):3842–7. doi: 10.1002/cncr.24440 19472396

[3] Tefferi A Vardiman JW. Classification and diagnosis of myeloproliferative neoplasms: the 2008 world health organization criteria and point-of-care diagnostic algorithms. Leukemia (2008) 22(1):14–22. doi: 10.1038/sj.leu.2404955 17882280

[4] Smith CA Fan G. The saga of JAK2 mutations and translocations in hematologic disorders: pathogenesis, diagnostic and therapeutic prospects, and revised world health organization diagnostic criteria for myeloproliferative neoplasms. Hum Pathol (2008) 39(6):795–810. doi: 10.1016/j.humpath.2008.02.004 18538168

[5] Orazi A Germing U. The myelodysplastic/myeloproliferative neoplasms: myeloproliferative diseases with dysplastic features. Leukemia (2008) 22(7):1308–19. doi: 10.1038/leu.2008.119 18480833

[6] Hoffman R Prchal JT Samuelson S Ciurea SO Rondelli D. Philadelphia Chromosome-negative myeloproliferative disorders: biology and treatment. Biol Blood Marrow Transplant (2007) 13(1 Suppl 1):64–72. doi: 10.1016/j.bbmt.2006.11.003 17222772

[7] Grinfeld J Nangalia J Green AR. Molecular determinants of pathogenesis and clinical phenotype in myeloproliferative neoplasms. Haematologica (2017) 102(1):7–17. doi: 10.3324/haematol.2014.113845 27909216 PMC5210228

[8] Constantinescu SN Vainchenker W Levy G Papadopoulos N. Functional consequences of mutations in myeloproliferative neoplasms. Hemasphere (2021) 5(6):e578. doi: 10.1097/HS9.0000000000000578 34095761 PMC8171364

[9] Levine RL Wadleigh M Cools J Ebert BL Wernig G Huntly BJ. Activating mutation in the tyrosine kinase JAK2 in polycythemia vera, essential thrombocythemia, and myeloid metaplasia with myelofibrosis. Cancer Cell (2005) 7(4):387–97. doi: 10.1016/j.ccr.2005.03.023 15837627

[10] Kralovics R Passamonti F Buser AS Teo SS Tiedt R Passweg JR. A gain-of-function mutation of JAK2 in myeloproliferative disorders. N Engl J Med (2005) 352(17):1779–90. doi: 10.1056/NEJMoa051113 15858187

[11] James C Ugo V Le Couedic JP Staerk J Delhommeau F Lacout C. A unique clonal JAK2 mutation leading to constitutive signalling causes polycythaemia vera. Nature (2005) 434(7037):1144–8. doi: 10.1038/nature03546 15793561

[12] Larsen TS Christensen JH Hasselbalch HC Pallisgaard N. The JAK2 V617F mutation involves b- and T-lymphocyte lineages in a subgroup of patients with Philadelphia-chromosome negative chronic myeloproliferative disorders. Br J Haematol (2007) 136(5):745–51. doi: 10.1111/j.1365-2141.2007.06497.x 17313377

[13] Zhang Y Liu L Jin L Yi X Dang E Yang Y. Oxidative stress-induced calreticulin expression and translocation: new insights into the destruction of melanocytes. J Invest Dermatol (2014) 134(1):183–91. doi: 10.1038/jid.2013.268 23771121

[14] Yu B Choi B Li W Kim DH. Magnetic field boosted ferroptosis-like cell death and responsive MRI using hybrid vesicles for cancer immunotherapy. Nat Commun (2020) 11(1):3637. doi: 10.1038/s41467-020-17380-5 32686685 PMC7371635

[15] Song S Lee JY Ermolenko L Mazumder A Ji S Ryu H. Tetrahydrobenzimidazole TMQ0153 triggers apoptosis, autophagy and necroptosis crosstalk in chronic myeloid leukemia. Cell Death Dis (2020) 11(2):109. doi: 10.1038/s41419-020-2304-8 32034134 PMC7007439

[16] Ikezaki M Minakata S Nishitsuji K Tabata S Lee Matsui IS Takatani M. Calreticulin protects insulin against reductive stress in vitro and in MIN6 cells. Biochimie (2020) 171-172:1–11. doi: 10.1016/j.biochi.2020.01.011 32004653

[17] Michalak M Corbett EF Mesaeli N Nakamura K Opas M. Calreticulin: one protein, one gene, many functions. Biochem J (1999) 344(Pt 2):281–92. doi: 10.1042/bj3440281 PMC1220642 10567207

[18] Wang WA Groenendyk J Michalak M. Calreticulin signaling in health and disease. Int J Biochem Cell Biol (2012) 44(6):842–6. doi: 10.1016/j.biocel.2012.02.009 22373697

[19] Houen G Hojrup P Ciplys E Gaboriaud C Slibinskas R. Structural analysis of calreticulin, an endoplasmic reticulum-resident molecular chaperone. Prog Mol Subcell Biol (2021) 59:13–25. doi: 10.1007/978-3-030-67696-4_2 34050860

[20] Danilczyk UG Cohen-Doyle MF Williams DB. Functional relationship between calreticulin, calnexin, and the endoplasmic reticulum luminal domain of calnexin. J Biol Chem (2000) 275(17):13089–97. doi: 10.1074/jbc.275.17.13089 10777614

[21] Ihara Y Urata Y Goto S Kondo T. Role of calreticulin in the sensitivity of myocardiac H9c2 cells to oxidative stress caused by hydrogen peroxide. Am J Physiol Cell Physiol (2006) 290(1):C208–21. doi: 10.1152/ajpcell.00075.2005 16135540

[22] Merlinsky TR Levine RL Pronier E. Unfolding the role of calreticulin in myeloproliferative neoplasm pathogenesis. Clin Cancer Res (2019) 25(10):2956–62. doi: 10.1158/1078-0432.CCR-18-3777 PMC6522317 30655313

[23] How J Hobbs GS Mullally A. Mutant calreticulin in myeloproliferative neoplasms. Blood (2019) 134(25):2242–8. doi: 10.1182/blood.2019000622 PMC6923668 31562135

[24] Prins D Gonzalez Arias C Klampfl T Grinfeld J Green AR. Mutant calreticulin in the myeloproliferative neoplasms. Hemasphere (2020) 4(1):e333. doi: 10.1097/HS9.0000000000000333 32382708 PMC7000472

[25] How J Garcia JS Mullally A. Biology and therapeutic targeting of molecular mechanisms in MPN. Blood (2022) 19:blood.2022017416. doi: 10.1182/blood.2022017416 PMC10163317 36534936

[26] Cazzola M Kralovics R. From janus kinase 2 to calreticulin: the clinically relevant genomic landscape of myeloproliferative neoplasms. Blood (2014) 123(24):3714–9. doi: 10.1182/blood-2014-03-530865 24786775

[27] Khalid F Damlaj M AlZahrani M Abuelgasim KA Gmati GE. Reactivation of tuberculosis following ruxolitinib therapy for primary myelofibrosis: Case series and literature review. Hematol Oncol Stem Cell Ther (2021) 14(3):252–6. doi: 10.1016/j.hemonc.2020.02.003 32201152

[28] Pietra D Rumi E Ferretti VV Di Buduo CA Milanesi C Cavalloni C. Differential clinical effects of different mutation subtypes in CALR-mutant myeloproliferative neoplasms. Leukemia (2016) 30(2):431–8. doi: 10.1038/leu.2015.277 PMC4740452 26449662

[29] Mesa RA Li CY Ketterling RP Schroeder GS Knudson RA Tefferi A. Leukemic transformation in myelofibrosis with myeloid metaplasia: a single-institution experience with 91 cases. Blood (2005) 105(3):973–7. doi: 10.1182/blood-2004-07-2864 15388582

[30] Scherer A Graff JM. Calmodulin differentially modulates Smad1 and Smad2 signaling. J Biol Chem (2000) 275(52):41430–8. doi: 10.1074/jbc.M005727200 11007779

[31] Lu M Xia L Liu YC Hochman T Bizzari L Aruch D. Lipocalin produced by myelofibrosis cells affects the fate of both hematopoietic and marrow microenvironmental cells. Blood (2015) 126(8):972–82. doi: 10.1182/blood-2014-12-618595 PMC4543230 26022238

[32] Martinaud C Desterke C Konopacki J Pieri L Torossian F Golub R. Osteogenic potential of mesenchymal stromal cells contributes to primary myelofibrosis. Cancer Res (2015) 75(22):4753–65. doi: 10.1158/0008-5472.CAN-14-3696 26404004

[33] Heck DE Shakarjian M Kim HD Laskin JD Vetrano AM. Mechanisms of oxidant generation by catalase. Ann N Y Acad Sci (2010) 1203:120–5. doi: 10.1111/j.1749-6632.2010.05603.x PMC4610122 20716293

[34] Hasselbalch HC. Chronic inflammation as a promotor of mutagenesis in essential thrombocythemia, polycythemia vera and myelofibrosis. a human inflammation model for cancer development? Leuk Res (2013) 37(2):214–20. doi: 10.1016/j.leukres.2012.10.020 23174192

[35] Mannino G Vicario N Parenti R Giuffrida R Lo Furno D. Connexin expression decreases during adipogenic differentiation of human adipose-derived mesenchymal stem cells. Mol Biol Rep (2020) 47(12):9951–8. doi: 10.1007/s11033-020-05950-1 33141287

[36] Musumeci G Lo Furno D Loreto C Giuffrida R Caggia S Leonardi R. Mesenchymal stem cells from adipose tissue which have been differentiated into chondrocytes in three-dimensional culture express lubricin. Exp Biol Med (Maywood) (2011) 236(11):1333–41. doi: 10.1258/ebm.2011.011183 22036733

[37] Lo Furno D Mannino G Giuffrida R. Functional role of mesenchymal stem cells in the treatment of chronic neurodegenerative diseases. J Cell Physiol (2018) 233(5):3982–99. doi: 10.1002/jcp.26192 28926091

[38] Giallongo C Tibullo D Camiolo G Parrinello NL Romano A Puglisi F. TLR4 signaling drives mesenchymal stromal cells commitment to promote tumor microenvironment transformation in multiple myeloma. Cell Death Dis (2019) 10(10):704. doi: 10.1038/s41419-019-1959-5 31541083 PMC6754430

[39] Goloviznina NA Verghese SC Yoon YM Taratula O Marks DL Kurre P. Mesenchymal stromal cell-derived extracellular vesicles promote myeloid-biased multipotent hematopoietic progenitor expansion *via* toll-like receptor engagement. J Biol Chem (2016) 291(47):24607–17. doi: 10.1074/jbc.M116.745653 PMC5114412 27758863

[40] Allegra A Pioggia G Tonacci A Casciaro M Musolino C Gangemi S. Synergic crosstalk between inflammation, oxidative stress, and genomic alterations in BCR-ABL-Negative myeloproliferative neoplasm. Antioxidants (Basel) (2020) 9(11):1037. doi: 10.3390/antiox9111037 33114087 PMC7690801

[41] Kile BT Alexander WS. The suppressors of cytokine signalling (SOCS). Cell Mol Life Sci (2001) 58(11):1627–35. doi: 10.1007/PL00000801 PMC11337286 11706989

[42] Palmer DC Restifo NP. Suppressors of cytokine signaling (SOCS) in T cell differentiation, maturation, and function. Trends Immunol (2009) 30(12):592–602. doi: 10.1016/j.it.2009.09.009 19879803 PMC2787651

[43] Forte D Garcia-Fernandez M Sanchez-Aguilera A Stavropoulou V Fielding C Martin-Perez D. Bone marrow mesenchymal stem cells support acute myeloid leukemia bioenergetics and enhance antioxidant defense and escape from chemotherapy. Cell Metab (2020) 32(5):829–43.e9. doi: 10.1016/j.cmet.2020.09.001 32966766 PMC7658808

[44] Quattrone F Dini V Barbanera S Zerbinati N Romanelli M. Cutaneous ulcers associated with hydroxyurea therapy. J Tissue Viability (2013) 22(4):112–21. doi: 10.1016/j.jtv.2013.08.002 24050921

[45] Ostojic A Vrhovac R Verstovsek S. Ruxolitinib for the treatment of myelofibrosis: its clinical potential. Ther Clin Risk Manag (2012) 8:95–103. doi: 10.2147/TCRM.S23277 22399854 PMC3295626

[46] Bjorn ME Brimnes MK Gudbrandsdottir S Andersen CL Poulsen HE Henriksen T. Ruxolitinib treatment reduces monocytic superoxide radical formation without affecting hydrogen peroxide formation or systemic oxidative nucleoside damage in myelofibrosis. Leuk Lymphoma (2019) 60(10):2549–57. doi: 10.1080/10428194.2019.1579323 30785365

[47] Yao JC Oetjen KA Wang T Xu H Abou-Ezzi G Krambs JR. TGF-beta signaling in myeloproliferative neoplasms contributes to myelofibrosis without disrupting the hematopoietic niche. J Clin Invest (2022) 132(11):e154092. doi: 10.1172/JCI154092 35439167 PMC9151699

[48] Teodorescu P Pasca S Jurj A Gafencu G Joelsson JP Selicean S. Transforming growth factor beta-mediated micromechanics modulates disease progression in primary myelofibrosis. J Cell Mol Med (2020) 24(19):11100–10. doi: 10.1111/jcmm.15526 PMC7576271 32889753

[49] Rossi C Zini R Rontauroli S Ruberti S Prudente Z Barbieri G. Role of TGF-beta1/miR-382-5p/SOD2 axis in the induction of oxidative stress in CD34+ cells from primary myelofibrosis. Mol Oncol (2018) 12(12):2102–23. doi: 10.1002/1878-0261.12387 PMC6275274 30259659

[50] Baryawno N Przybylski D Kowalczyk MS Kfoury Y Severe N Gustafsson K. A cellular taxonomy of the bone marrow stroma in homeostasis and leukemia. Cell (2019) 177(7):1915–32.e16. doi: 10.1016/j.cell.2019.04.040 31130381 PMC6570562

[51] Tikhonova AN Dolgalev I Hu H Sivaraj KK Hoxha E Cuesta-Dominguez A. The bone marrow microenvironment at single-cell resolution. Nature (2019) 569(7755):222–8. doi: 10.1038/s41586-019-1104-8 PMC6607432 30971824

[52] Malara A Abbonante V Zingariello M Migliaccio A Balduini A. Megakaryocyte contribution to bone marrow fibrosis: many arrows in the quiver. Mediterr J Hematol Infect Dis (2018) 10(1):e2018068. doi: 10.4084/mjhid.2018.068 30416700 PMC6223581

[53] Leimkuhler NB Gleitz HFE Ronghui L Snoeren IAM Fuchs SNR Nagai JS. Heterogeneous bone-marrow stromal progenitors drive myelofibrosis *via* a druggable alarmin axis. Cell Stem Cell (2021) 28(4):637–52.e8. doi: 10.1016/j.stem.2020.11.004 33301706 PMC8024900

[54] Decker M Martinez-Morentin L Wang G Lee Y Liu Q Leslie J. Leptin-receptor-expressing bone marrow stromal cells are myofibroblasts in primary myelofibrosis. Nat Cell Biol (2017) 19(6):677–88. doi: 10.1038/ncb3530 PMC5801040 28481328

[55] Reinhardt JW Breuer CK. Fibrocytes: A critical review and practical guide. Front Immunol (2021) 12:784401. doi: 10.3389/fimmu.2021.784401 34975874 PMC8718395

[56] Sapudom J Muller CD Nguyen KT Martin S Anderegg U Pompe T. Matrix remodeling and hyaluronan production by myofibroblasts and cancer-associated fibroblasts in 3D collagen matrices. Gels (2020) 6(4):33. doi: 10.3390/gels6040033 33008082 PMC7709683

[57] Tibullo D Longo A Vicario N Romano A Barbato A Di Rosa M. Ixazomib improves bone remodeling and counteracts sonic hedgehog signaling inhibition mediated by myeloma cells. Cancers (Basel) (2020) 12(2):323. doi: 10.3390/cancers12020323 32019102 PMC7073172

[58] Longhitano L Tibullo D Vicario N Giallongo C La Spina E Romano A. IGFBP-6/sonic hedgehog/TLR4 signalling axis drives bone marrow fibrotic transformation in primary myelofibrosis. Aging (Albany NY) (2021) 13(23):25055–71. doi: 10.18632/aging.203779 PMC8714138 34905501

[59] Zahr AA Salama ME Carreau N Tremblay D Verstovsek S Mesa R. Bone marrow fibrosis in myelofibrosis: pathogenesis, prognosis and targeted strategies. Haematologica (2016) 101(6):660–71. doi: 10.3324/haematol.2015.141283 PMC5013940 27252511

[60] Schneider RK Mullally A Dugourd A Peisker F Hoogenboezem R Van Strien PMH. Gli1(+) mesenchymal stromal cells are a key driver of bone marrow fibrosis and an important cellular therapeutic target. Cell Stem Cell (2017) 20(6):785–800.e8. doi: 10.1016/j.stem.2017.03.008 28457748 PMC6485654

[61] van Bon L Affandi AJ Broen J Christmann RB Marijnissen RJ Stawski L. Proteome-wide analysis and CXCL4 as a biomarker in systemic sclerosis. N Engl J Med (2014) 370(5):433–43. doi: 10.1056/NEJMoa1114576 PMC4040466 24350901

[62] Kramann R Schneider RK. The identification of fibrosis-driving myofibroblast precursors reveals new therapeutic avenues in myelofibrosis. Blood (2018) 131(19):2111–9. doi: 10.1182/blood-2018-02-834820 29572380

[63] Arranz L Sanchez-Aguilera A Martin-Perez D Isern J Langa X Tzankov A. Neuropathy of haematopoietic stem cell niche is essential for myeloproliferative neoplasms. Nature (2014) 512(7512):78–81. doi: 10.1038/nature13383 25043017

[64] Torrisi F Alberghina C D'Aprile S Pavone AM Longhitano L Giallongo S. The hallmarks of glioblastoma: Heterogeneity, intercellular crosstalk and molecular signature of invasiveness and progression. Biomedicines (2022) 10(4):806. doi: 10.3390/biomedicines10040806 35453557 PMC9031586

[65] Camiolo G Barbato A Giallongo C Vicario N Romano A Parrinello NL. Iron regulates myeloma cell/macrophage interaction and drives resistance to bortezomib. Redox Biol (2020) 36:101611. doi: 10.1016/j.redox.2020.101611 32863212 PMC7327252

[66] Spampinato M Giallongo C Romano A Longhitano L La Spina E Avola R. Focus on osteosclerotic progression in primary myelofibrosis. Biomolecules (2021) 11(1):122. doi: 10.3390/biom11010122 33477816 PMC7832894

[67] Osterhoff G Morgan EF Shefelbine SJ Karim L McNamara LM Augat P. Bone mechanical properties and changes with osteoporosis. Injury (2016) 47 Suppl 2(Suppl 2):S11–20. doi: 10.1016/S0020-1383(16)47003-8 PMC4955555 27338221

[68] Morrison SJ Scadden DT. The bone marrow niche for haematopoietic stem cells. Nature (2014) 505(7483):327–34. doi: 10.1038/nature12984 PMC4514480 24429631

[69] Sacchetti B Funari A Michienzi S Di Cesare S Piersanti S Saggio I. Self-renewing osteoprogenitors in bone marrow sinusoids can organize a hematopoietic microenvironment. Cell (2007) 131(2):324–36. doi: 10.1016/j.cell.2007.08.025 17956733

[70] Mannino G Russo C Maugeri G Musumeci G Vicario N Tibullo D. Adult stem cell niches for tissue homeostasis. J Cell Physiol (2022) 237(1):239–57. doi: 10.1002/jcp.30562 PMC9291197 34435361

[71] Musumeci G Mobasheri A Trovato FM Szychlinska MA Graziano AC Lo Furno D. Biosynthesis of collagen I, II, RUNX2 and lubricin at different time points of chondrogenic differentiation in a 3D *in vitro* model of human mesenchymal stem cells derived from adipose tissue. Acta Histochem (2014) 116(8):1407–17. doi: 10.1016/j.acthis.2014.09.008 25307495

[72] Glenn JD Whartenby KA. Mesenchymal stem cells: Emerging mechanisms of immunomodulation and therapy. World J Stem Cells (2014) 6(5):526–39. doi: 10.4252/wjsc.v6.i5.526 PMC4178253 25426250

[73] Park D Spencer JA Koh BI Kobayashi T Fujisaki J Clemens TL. Endogenous bone marrow MSCs are dynamic, fate-restricted participants in bone maintenance and regeneration. Cell Stem Cell (2012) 10(3):259–72. doi: 10.1016/j.stem.2012.02.003 PMC3652251 22385654

[74] Veletic I Manshouri T Multani AS Yin CC Chen L Verstovsek S. Myelofibrosis osteoclasts are clonal and functionally impaired. Blood (2019) 133(21):2320–4. doi: 10.1182/blood-2018-10-878926 PMC6533604 30745304

[75] Schepers K Pietras EM Reynaud D Flach J Binnewies M Garg T. Myeloproliferative neoplasia remodels the endosteal bone marrow niche into a self-reinforcing leukemic niche. Cell Stem Cell (2013) 13(3):285–99. doi: 10.1016/j.stem.2013.06.009 PMC3769504 23850243

[76] Desterke C Martinaud C Ruzehaji N Le Bousse-Kerdiles MC. Inflammation as a keystone of bone marrow stroma alterations in primary myelofibrosis. Mediators Inflammation (2015) 2015:415024. doi: 10.1155/2015/415024 PMC4660030 26640324

[77] Fisher DAC Fowles JS Zhou A Oh ST. Inflammatory pathophysiology as a contributor to myeloproliferative neoplasms. Front Immunol (2021) 12:683401. doi: 10.3389/fimmu.2021.683401 34140953 PMC8204249

[78] Ramundo V Giribaldi G Aldieri E. Transforming growth factor-beta and oxidative stress in cancer: A crosstalk in driving tumor transformation. Cancers (Basel) (2021) 13(12):3093. doi: 10.3390/cancers13123093 34205678 PMC8235010

[79] Fedeles BI Freudenthal BD Yau E Singh V Chang SC Li D. Intrinsic mutagenic properties of 5-chlorocytosine: A mechanistic connection between chronic inflammation and cancer. Proc Natl Acad Sci U.S.A. (2015) 112(33):E4571–80. doi: 10.1073/pnas.1507709112 PMC4547254 26243878

[80] Knutson CG Mangerich A Zeng Y Raczynski AR Liberman RG Kang P. Chemical and cytokine features of innate immunity characterize serum and tissue profiles in inflammatory bowel disease. Proc Natl Acad Sci U.S.A. (2013) 110(26):E2332–41. doi: 10.1073/pnas.1222669110 PMC3696795 23754421

[81] Mangerich A Knutson CG Parry NM Muthupalani S Ye W Prestwich E. Infection-induced colitis in mice causes dynamic and tissue-specific changes in stress response and DNA damage leading to colon cancer. Proc Natl Acad Sci U S A (2012) 109(27):E1820–9. doi: 10.1073/pnas.1207829109 PMC3390855 22689960

[82] Jin B Robertson KD. DNA Methyltransferases, DNA damage repair, and cancer. Adv Exp Med Biol (2013) 754:3–29. doi: 10.1007/978-1-4419-9967-2_1 22956494 PMC3707278

[83] Giallongo S Lo Re O Lochmanova G Parca L Petrizzelli F Zdrahal Z. Phosphorylation within intrinsic disordered region discriminates histone variant macroH2A1 splicing isoforms-macroH2A1.1 and macroH2A1.2. Biol (Basel) (2021) 10(7):659. doi: 10.3390/biology10070659 PMC8301376 34356514

[84] Giallongo S Longhitano L Denaro S D'Aprile S Torrisi F La Spina E. The role of epigenetics in neuroinflammatory-driven diseases. Int J Mol Sci (2022) 23(23):15218. doi: 10.3390/ijms232315218 36499544 PMC9740629

[85] Van den Worm E Beukelman CJ Van den Berg AJ Kroes BH Labadie RP Van Dijk H. Effects of methoxylation of apocynin and analogs on the inhibition of reactive oxygen species production by stimulated human neutrophils. Eur J Pharmacol (2001) 433(2-3):225–30. doi: 10.1016/S0014-2999(01)01516-3 11755156

[86] Valinluck V Liu P Kang JI Jr. Burdzy A Sowers LC. 5-halogenated pyrimidine lesions within a CpG sequence context mimic 5-methylcytosine by enhancing the binding of the methyl-CpG-binding domain of methyl-CpG-binding protein 2 (MeCP2). Nucleic Acids Res (2005) 33(9):3057–64. doi: 10.1093/nar/gki612 PMC1140371 15917437

[87] Checa J Aran JM. Reactive oxygen species: Drivers of physiological and pathological processes. J Inflammation Res (2020) 13:1057–73. doi: 10.2147/JIR.S275595 PMC7719303 33293849

[88] Haas S Hansson J Klimmeck D Loeffler D Velten L Uckelmann H. Inflammation-induced emergency megakaryopoiesis driven by hematopoietic stem cell-like megakaryocyte progenitors. Cell Stem Cell (2015) 17(4):422–34. doi: 10.1016/j.stem.2015.07.007 26299573

[89] Di Veroli A Campagna A De Muro M Maurillo L Trawinska MM LeonettiCrescenzi S. Deferasirox in the treatment of iron overload during myeloproliferative neoplasms in fibrotic phase: does ferritin decrement matter? Leuk Res (2019) 76:65–9. doi: 10.1016/j.leukres.2018.11.012 30578958

[90] Mendez Luque LF Blackmon AL Ramanathan G Fleischman AG. Key role of inflammation in myeloproliferative neoplasms: Instigator of disease initiation, progression. and symptoms. Curr Hematol Malig Rep (2019) 14(3):145–53. doi: 10.1007/s11899-019-00508-w PMC7746200 31119475

